# Disease management programs in heart failure: half a century of an unmet need

**DOI:** 10.1007/s00508-017-1286-8

**Published:** 2017-11-14

**Authors:** Deddo Moertl

**Affiliations:** 1 Clinical Department of Internal Medicine III, University Hospital St. Poelten, Karl Landsteiner University of Health Sciences, Propst Fuehrer-Straße 4, 3100 St. Poelten, Austria; 2 Karl Landsteiner Institute for the Research of Ischemic Cardiac Diseases and Rhythmology, St. Poelten, Austria

Heart failure (HF) is a devastating disease with severe symptoms, reduced quality of life and a poor prognosis. Without appropriate treatment, HF has a higher mortality than many forms of cancer [1]. Hospitalization and readmission are common, which negatively affect patient quality of life and cause considerable health-economic costs [2].

Fortunately, there are good treatment options for chronic HF, in particular for patients with a reduced left ventricular ejection fraction. Disease-modifying therapies including angiotensin-converting enzyme (ACE) inhibitors, beta-adrenergic blockers, mineralocorticoid receptor blockers, cardiac resynchronization therapy and the recently marketed sacubitril/valsartan showed positive effects on exercise capacity and quality of life as well as reducing hospitalization and mortality rates [3]. Just with appropriate drug treatment we can triple the patients’ remaining life span [4].

Despite these overwhelming benefits shown in clinical trials, guideline-recommended treatment is not delivered to the real-world patient. Data from 13 Austrian health insurance funds analyzing >36,000 patients showed a disastrous drug adherence after discharge from HF hospitalization. After a median of 614 days, prescriptions for ACE inhibitors, beta-adrenergic blockers, and mineralocorticoid receptor-antagonists were filled in only 49.3%, 40.4%, and 16.1% of the cases, respectively [5]. Thus, the question arises how implementation of HF guidelines can be improved. One option is the inclusion of HF patients in a multidisciplinary disease management program (DMP), which has received a class I recommendation in international HF guidelines for more than 10 years [3, 6]. Such programs can improve patient well-being, reduce hospitalizations and prevent premature death [7]. Beyond these clinical benefits, these programs have proven to be cost-effective and some even cost-saving [8]; however, apart from local initiatives with a high variety in design, duration, size and effect, no nationwide structured HF service has so far been implemented in Austria. Therefore, the Heart Failure Working Group of the Austrian Society of Cardiology has decided to elaborate a position paper on DMPs for chronic HF within the Austrian context. This position paper aims to provide evidence-based arguments for the need and efficacy of a comprehensive DMP for HF and to describe the essential components of such a program. It is based on the recommendations of the European Society of Cardiology (ESC) [9], which were adapted to Austrian circumstances. The key elements are multidisciplinary treatment including physician and nurse HF specialists, a seamless integration of all sectors of care from primary care to tertiary centers, HF outpatient clinics serving as expert referral centers for the entire network, and adherence to guidelines. Integrated HF management within a DMP needs to be coordinated, which in most models is done by a specialist HF nurse. Other tasks of this nurse can be implementation of home visits, supportive monitoring of treatment optimization, early recognition of worsening HF, and facilitation of patient empowerment.

While this position paper has been urgently needed and its importance for the promotion of guideline-recommended treatment in Austria is obvious, it must be noted that the demands made are not new. For example, in an article published in the American Journal of Public Health in the 1960s entitled “Congestive heart failure, the patient and the community”, Raymond T. Benack already outlined a DMP for chronic HF [10]. Accounting for the huge number of patients, their complexity and poor prognosis he recommended a multidisciplinary approach. Nurses trained in HF should perform home visits, check physical status and adherence to recommendations, and report to the treating physician. For Benack the main benefit of home-based nursing care for HF was a reduction in hospitalization, mainly achieved by early detection of worsening HF. Thus, he concluded “Through these combined services the patient will receive better home care, recurrences of congestive heart failure can be controlled, and the community and the patient will show a financial saving through decreased hospital readmissions and decreased total hospital time. Finally, of course, the patient will enjoy a longer and more useful life because of the prevention of complications from repeated attacks of congestive heart failure.”

Despite being published in 1964, the basic characteristics of a DMP for HF as described by Benack are still valid today and in the main do not differ from the positions presented in the Austrian paper [11] or the corresponding ESC statement [9]. While the cardiac community is often considered to be a fast adopter of innovations, at least when we think of interventional technologies, the lack of DMPs seems even more striking considering that Benack’s paper was published more than half a century ago and randomized controlled trials demonstrating the benefits of DMPs date back to the 1990s [12]. Obviously, there is some ongoing inertia of the responsible healthcare authorities to implement this class I A recommendation for decades, and the reasons for this impressive example of non-implementation of evidence-based medicine are definitely complex and hard to understand.

## Where do we go from here?

The position paper makes a strong claim for the nationwide implementation of structured HF management; however, is it necessary to implement a one-size-fits-all program for all regions? Probably not. Some diversity in design of DMPs for HF will be acceptable, provided certain characteristics and components are implemented and the clinical benefit and the cost-effectiveness is guaranteed and regularly scrutinized. The next step should be the evaluation of ongoing DMPs according to the requirements given in the position paper. Quality control and regular audits as integrated parts of a DMP will be essential items for evaluation, giving insights into efficacy and cost-effectiveness of specific DMPs.

Regions without DMPs for HF should implement one as soon as possible and the presented position paper could serve as a blueprint; however, implementation should not rely on the ambitious initiatives of local opinion leaders trying to raise funds via various sources. Since the evidence for the clinical and economic benefit is clear and given a class IA recommendation in the European guidelines, it is the urgent responsibility of the official healthcare authorities to establish and run DMPs for HF.
